# Journey Through Words: Exploring Esophageal Cancer in Literature

**DOI:** 10.7759/cureus.48411

**Published:** 2023-11-06

**Authors:** M Hasaan Shahid, Reda H Mithany, Samana Aslam, Nesma Daniel, Farid Gerges, Muhammad Umar Gill, Andrew Wanees, Shenouda Abdallah, Mark Abdelmaseeh, Abdul Hannan

**Affiliations:** 1 Surgery, Glangwili General Hospital, Carmarthen, GBR; 2 Laparoscopic Colorectal Surgery, Kingston Hospital National Health Service (NHS) Foundation Trust, Kingston, GBR; 3 General Surgery, Lahore General Hospital, Lahore, PAK; 4 Medical Laboratory Science, Ain Shams University, Cairo, EGY; 5 General and Emergency Surgery, Kingston Hospital National Health Service (NHS) Foundation Trust, Kingston, GBR; 6 Accident and Emergency, King's College Hospital National Health Service (NHS) Foundation Trust, London, GBR; 7 General Surgery, Dar El-Salam General Hospital, Cairo, EGY; 8 Surgery, Jaber Al-Ahmad Hospital, Kuwait City, KWT; 9 General Surgery, Faculty of Medicine, Assuit University, Assuit, EGY

**Keywords:** emerging therapies, advancements, recurrence, treatment modalities, staging, diagnosis, risk factors, adenocarcinoma, squamous cell carcinoma, esophageal cancer

## Abstract

Esophageal cancer is a significant global health challenge, characterized by its aggressive nature and high mortality rates. The disease disproportionately affects males and ranks among the leading causes of cancer-related deaths worldwide. Alarming projections indicate that the prevalence of esophageal cancer is expected to surge by approximately 140% by the year 2025. This trend starkly contrasts with the anticipated decline in incidence observed for many other types of cancers. The cancer manifests primarily in two major subtypes: esophageal squamous cell carcinoma and adenocarcinoma, each with distinct epidemiological and biological characteristics. This review provides an in-depth exploration of the risk factors, anatomy, clinical presentation, diagnosis, staging, prognosis, treatment modalities, recurrence, advancements, and emerging therapies in esophageal cancer. Additionally, preventive and early detection strategies are discussed, focusing on primary, secondary, and tertiary prevention approaches. A comprehensive understanding of esophageal cancer is vital for formulating effective management strategies and improving patient outcomes.

## Introduction and background

Esophageal cancer represents a significant health concern globally, particularly due to its aggressive nature and substantial mortality rates. It is a malignancy that disproportionately affects males and stands as a prominent contributor to cancer-related fatalities on a global scale. In the year 2005 alone, esophageal cancer accounted for more than 400,000 deaths worldwide, positioning it as the eighth most prevalent cancer and the sixth leading cause of cancer-related deaths internationally. Notably, a vast majority of esophageal cancer cases and related deaths, exceeding 80%, were concentrated in developing nations [1]. Furthermore, disconcerting trends portend a projected escalation in the prevalence of esophageal cancer by approximately 140% by the year 2025. This stands in stark contrast to the expected decline in incidence for numerous other types of cancers [2].

Esophageal cancer manifests primarily in two major subtypes: esophageal squamous cell carcinoma (ESCC) and esophageal adenocarcinoma (EAC). These subtypes possess distinct epidemiological and biological characteristics, necessitating a comprehensive understanding of their differences for effective management and treatment [3].

## Review

Anatomy of the esophagus

**Figure 1:**
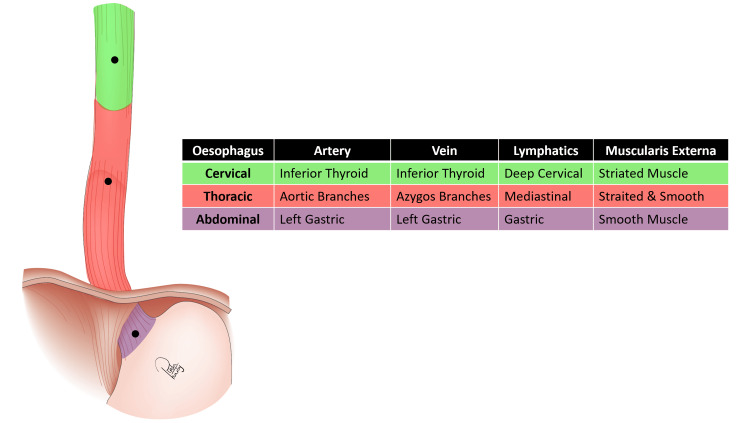
The Esophageal Parts Anatomy This medical illustration has been drawn by Reda Harby, one of the authors.

The esophagus serves as a direct continuation of the pharynx, establishing a vital connection to the stomach (Figure 1). The esophagus is characterized by its muscular tube structure, which typically measures between 25 and 30 centimeters in length in the average adult. The esophagus begins at the lower border of the cricoid cartilage in the neck. It then descends through the neck and extends into the thorax, passing through the superior and posterior mediastinum before penetrating the diaphragm to reach the abdominal cavity. For descriptive purposes, the esophagus is conventionally subdivided into three distinct segments: cervical, thoracic, and abdominal. The thoracic section predominates in length and can be further categorized into superior and posterior mediastinal segments [4].

Histological analysis

When viewed through a microscope, the esophagus displays identifiable layers characteristic of the digestive tract, including the mucosa, submucosa, muscularis propria, and adventitia. The mucosal lining of the esophagus consists entirely of non-keratinized, stratified squamous epithelium. At the point where the esophagus meets the stomach (the gastro-esophageal or esophagogastric junction), there is a noticeable transition in the type of epithelial lining. Here, the squamous epithelium of the esophagus is substituted by the columnar epithelium characteristic of the gastric mucosa. This specific histological zone, termed as the esophagogastric junction or 'Z-line,' is typically situated beneath the diaphragm. In the submucosal layer, particularly in the lower third of the esophagus, a few mucous glands are present, and their ducts open into the esophageal lumen. The secretions from these glands aid in the lubrication of solid food during ingestion. The muscularis propria comprises two layers of muscle: an inner circular layer and an outer longitudinal layer. In the upper third of the esophagus, the muscularis propria is solely composed of striated (skeletal) voluntary muscle. In the lower third, it consists entirely of smooth (visceral) involuntary muscle. The middle third of the esophagus predominantly features smooth muscle in the muscularis propria, with a combination of striated muscle fibers. Surrounding the muscularis propria is an adventitial layer. In contrast to the majority of other parts of the gastrointestinal tract, the esophagus does not possess a serosal layer [4,5].

The course of the esophagus

Originating from the midline, the esophagus undergoes a slight leftward inclination in the lower neck region before progressing into the superior mediastinum along the midline. Gradually deviating and inclining to the left, it positions itself behind the left main bronchus, subsequently crossing to the right at the T6 vertebra level. Eventually, it crosses back to the left of the midline, anterior to the descending thoracic aorta, and punctuates its course by traversing the diaphragm at the T10 vertebra level. Notably, the normal esophagus manifests four points of constriction along its length, distinctly visible as indentations in radiological imaging via a 'barium swallow.' These constrictions are attributed to the upper esophageal sphincter (cricopharyngeus), the area of contact between the aortic arch and the left esophageal wall, the contact site with the left main bronchus, and the oesophageal hiatus in the diaphragm, respectively. In an average adult, these four constrictions are approximately located at 15 cm, 23 cm, 27 cm, and 39 cm from the incisor teeth [5,6].

Risk factors

Table 1 presents a summary of the key risk factors associated with esophageal carcinoma [7-10].

**Table 1:** Risk Factors Associated With Esophageal Carcinoma

Risk Factor	Description
Demographics	Age > 50 years White race (Adenocarcinoma) Black race (Squamous Cell Carcinoma) Male > Female
Smoking	Increased risk in current smokers and duration of smoking is important
Alcohol Consumption	Moderate to high intake
Gastroesophageal Reflux Disease (GERD)	Increased risk of development
Diet and Nutrition	Western diet low intake of fruits, vegetables, non-fried fish low levels of selenium, zinc, vitamin E and other micronutrient deficiencies
Obesity and Body Composition	Elevated BMI
Other Risk Factors	Achalasia and increased gastric acid secretions

The genetic factors associated with ESCC and EAC, providing a clear overview of their roles and associations within each subtype of esophageal carcinoma summarized in Table 2 [7,10,11].

**Table 2:** Genetic Factors Associated With Esophageal Cancers VEGF: vascular endothelial growth factor, ESCC: esophageal squamous cell carcinoma, EAC: esophageal adenocarcinoma

Genetic Factors	Description
Genes that Regulate the Cell Cycle or Differentiation	
TP53	>83% of ESCCs contain a somatic mutation in TP53.
CDKN2A, RB1, NFE2L2, CHEK1, CHEK2, NOTCH1, NOTCH3	Mutations in 2-10% of ESCCs.
Overexpressed Genes: CCND1, CDK4/CDK6, MDM2	Overexpressed in ESCCs.
BTG3	Decreased expression in tumor tissues, correlated with lymph node metastasis and tumor staging in EAC.
Epidermal Growth Factor Receptor (EGFR) and Receptor Tyrosine Kinase or RAS Signaling	
EGFR	Overexpressed in 59.6-76% of ESCCs, associated with poor prognosis.
Downstream Factors: Receptor tyrosine kinase, RAS, AKT pathways	Mutations or amplifications in 78.6% of ESCCs, associated with prognosis and clinical stage.
Genetic Factors in Vascular Endothelial Growth Factor Signal Pathway	
VEGF-C	Strong expression in 75% of tumor samples, correlated with tumor stages, lymph node metastasis, and survival in EAC.
Genetic Polymorphisms: FLT1 (rs3794405), VEGF 936 C > T polymorphism	Risk associations with mortality in esophageal adenocarcinoma, correlation with event-free survival.
Epigenetic Factors	
Epigenetic Alterations	DNA methylation, histone modification, loss of genome imprinting associated with ESCC development.
Methylation of Specific Genes: APC, RB1, CDKN2A	Detected in ESCC, promote ESCC progression.
Polymorphisms and Promoter Methylation: TP53, MDM21, CASP8, COX254, GPX7, cyclin E1 (CCNE1)	Associated with ESCC risk, influence risk of developing ESCC.

Clinical presentation and diagnosis

Esophageal carcinoma typically presents with dysphagia, a primary symptom that significantly impedes swallowing. This challenge is compounded by unexplained weight loss, signifying the gravity of the condition. Patients may also experience epigastric and back pain, further impacting their daily lives. These discomforts are often accompanied by sensations of pressure or burning in the chest or upper abdomen, heightening the overall distress. Additionally, individuals may report worsening indigestion or heartburn, along with persistent coughing or hoarseness, adding complexity to the clinical presentation [12,13].

At the time of initial diagnosis, approximately half of the patients present with distant metastasis, and subsequent to surgery or radiotherapy, more than one-third develop distant metastases. The development of distant metastases typically occurs within six months of radical treatment, with a median survival of only five months after diagnosis of distant metastasis. Hence, distant metastasis stands as a significant factor contributing to treatment failure and mortality in esophageal cancer. In a comprehensive study by Wu et al. involving 1,470 patients, various sites of distant metastasis were identified. The liver emerged as the most prevalent site of metastasis, observed in approximately 32.4% of cases, followed by distant lymph nodes (28.4%), lungs (20.5%), bones (15.3%), and brain (3.4%). Comprehending the specific locations of distant metastases is essential for gaining valuable insights into disease progression and forming effective treatment strategies [14,15].

**Figure 2:**
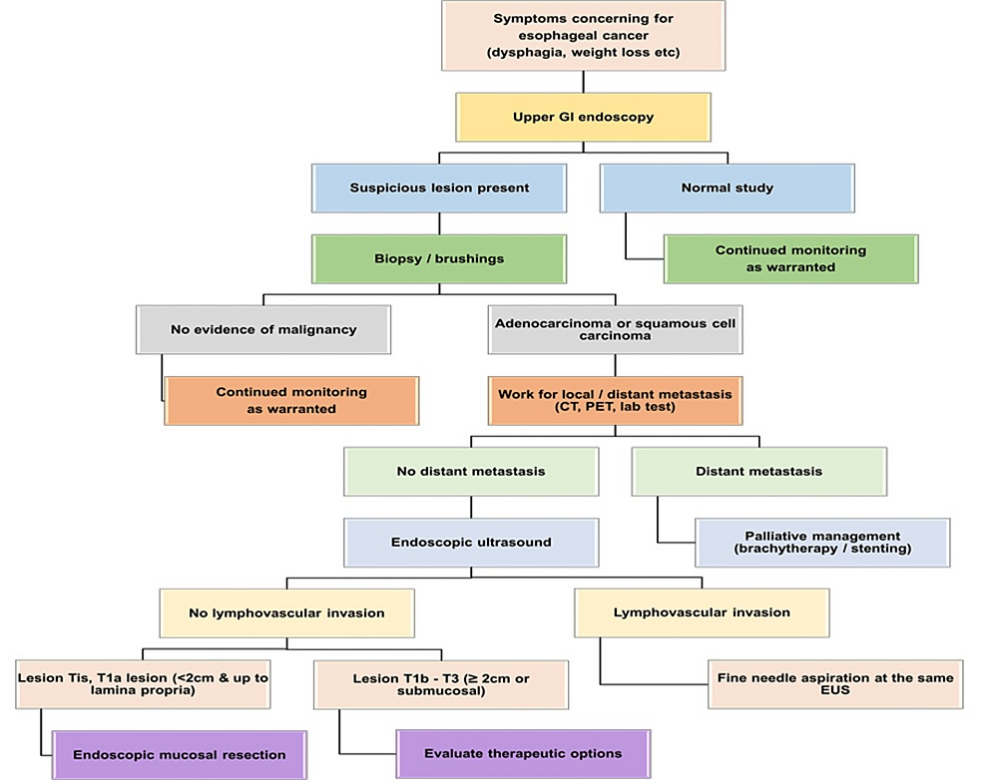
Algorithm for Workup for Symptoms Concerning Esophageal Cancer This figure is made by one of the authors. GI: gastrointestinal, EUS: endoscopic ultrasound

Both the Society of Thoracic Surgeons and the National Comprehensive Cancer Network (NCCN) recommend upper endoscopy as the initial diagnostic evaluation for individuals exhibiting the aforementioned clinical presentation to rule out esophageal cancer. Figure 2 demonstrates an algorithm for workup for symptoms concerning esophageal cancer [9].

Staging

Table 3 demonstrates the staging of esophageal carcinoma [16-18].

**Table 3:** Staging of Esophageal Carcinoma

T Category (Tumor)	
T Category	Description
TX	Tumor assessment not possible
T0	No primary tumor evidence
Tis	High-grade dysplasia, malignant cells confined by the basement membrane
T1	Invasion of lamina propria, muscularis mucosa, or submucosa
T1a	Invasion of lamina propria or muscularis mucosa
T1b	Invasion of submucosa
T2	Invasion of muscularis propria
T3	Invasion of adventitia
T4	Invasion of adjacent structures
T4a	Invasion of pleura, azygos vein, diaphragm, pericardium, or peritoneum (resectable structures)
T4b	Invasion of other (unresectable structures) adjacent structures (e.g., trachea, vertebral body, or aorta)

N Category (Regional Lymph Nodes)	
N Category	Description
NX	Regional lymph nodes assessment not possible
N0	No regional lymph node metastasis
N1	Metastasis in 1-2 regional lymph nodes
N2	Metastasis in 3-6 regional lymph nodes
N3	Metastasis in 7 or more regional lymph nodes

M Category (Distant Metastasis)	
M Category	Description
M0	No distant metastasis
M1	Distant metastasis

Treatment modalities for esophageal carcinoma

The management of esophageal carcinoma encompasses a range of approaches, each tailored to the specific characteristics and stage of the disease. Key treatment modalities include endoscopic therapy, surgery, ablation techniques, reconstruction conduits, systemic therapy, and radiation therapy [19-21].

Endoscopic Therapy

Endoscopic therapy utilizes specialized scopes to eliminate or eradicate early-stage esophageal cancer and Barrett's esophagus (BE). This encompassing approach incorporates procedures such as endoscopic mucosal resection (EMR), endoscopic submucosal dissection (ESD), and ablation techniques.

Surgery

Surgical intervention, notably esophagectomy, entails the partial or complete removal of the esophagus. This procedure can be executed through open surgery or less invasive techniques, including laparoscopic or thoracoscopic approaches. Reconstruction of the esophagus involves employing conduits like the stomach and large or small bowel to restore continuity after excision. The gastric conduit, derived from the stomach, is the prevalent choice for esophageal reconstruction.

Ablation

Ablation techniques employ various methods, including extreme cold, heat, micro and radio waves, and chemicals, to obliterate cancerous cells. Modalities like cryotherapy, radiofrequency ablation (RFA), and photodynamic therapy (PTD) fall within this category.

Other Procedures

Additional procedures, such as esophagogastrectomy, gastrectomy, lymph node dissection, and the insertion of gastrostomy tubes (G-tube) or jejunostomy tubes (J-tube), may be performed based on unique clinical circumstances.

Systemic Therapy

Systemic therapy encompasses a multifaceted approach, encompassing chemotherapy, targeted therapy, and immunotherapy, to combat cancer at a systemic level. It may be administered pre or post-surgery, or in advanced stages to alleviate symptoms and enhance quality of life.

Radiation Therapy

Radiation therapy employs high-energy radiation to eliminate cancer cells and reduce tumor size. The administration can be external (external beam radiation therapy - EBRT) or internal (brachytherapy). The selection of a specific treatment modality is contingent on critical factors including the stage and location of the cancer, as well as the overall health condition of the patient. Combinations of these therapeutic modalities are often deployed to attain the most favorable outcomes [19-21].

Table 4 displays a stage-wise classification and treatment segregation for esophageal cancer based on surgical fitness [11,19,22].

**Table 4:** Stage-Wise Classification and Treatment Segregation for Esophageal Cancer Based on Surgical Fitness

Stage	Tumor Description	Fit for Surgery Treatment	Unfit for Surgery Treatment
Squamous Cell Carcinoma			
Early (pTis)	Abnormal cells in the epithelium layer of mucosa	Endoscopic therapies (ER), ER followed by ablation, or ablation. Esophagectomy is also an option.	Endoscopic resection and/or ablation. Chemoradiation is an option.
Early (pT1a)	The tumor infiltrates the lamina propria or muscularis mucosa layer	Endoscopic resection (ER) or ER paired with ablation is preferred. Esophagectomy is also an option.	Endoscopic resection (ER) or ER followed by ablation is preferred. Chemoradiation may follow.
Early (pT1b)	Tumor invades submucosa	Esophagectomy is the option.	Endoscopic resection (ER) or ER followed by ablation is preferred. Chemoradiation might follow.
Locoregional (cT1b to T2, N0)	Tumor not in cervical esophagus, <3 cm, no lymph node involvement	Esophagectomy is an option.	Definitive chemoradiation or perioperative chemotherapy.
Locoregional (cT2 or node-positive to cT4a)	Tumor ≥3 cm, node-positive, or grown into the third layer of esophagus wall	Preoperative chemoradiation followed by esophagectomy (non-cervical esophagus), or definitive chemoradiation (cervical esophagus). After chemoradiation, further treatment based on disease status.	Definitive chemoradiation if not opting for esophagectomy, or palliative care.
Locoregional (cT4b)	Tumor grown through layers into nearby structures	Definitive chemoradiation, and esophagectomy if disease remains. Chemotherapy alone might be possible in specific cases. After chemoradiation, further treatment based on disease status.	Palliative radiation therapy or best supportive care.
Adenocarcinoma of Esophagus			
Early (pTis)	Abnormal cells	Esophagectomy could be considered as a potential treatment option. Chemoradiation is an alternative if surgery is not opted.	Endoscopic resection (ER), ER followed by ablation, or ablation are options. Chemoradiation is an option if patient is unfit for surgery.
Early (pT1a)	Tumor invades lamina propria or muscularis mucosa layer	Esophagectomy might be an option if the patient is fit for surgery. Chemoradiation is an option if surgery is not chosen.	Endoscopic resection (ER) or ER followed by ablation is preferred. Chemoradiation might follow if surgery is not an option.
Early (pT1b)	Tumor invades submucosa	ER followed by ablation or esophagectomy is recommended for superficial pT1b. Esophagectomy if node-negative.	ER followed by ablation is recommended. Chemoradiation if esophagectomy is not possible.
Locoregional (cT1b to T2, N0)	Tumor not in cervical esophagus, <3 cm, no lymph node involvement	Esophagectomy might be an option if the patient is fit for surgery. Chemoradiation is an option if surgery is not chosen.	Definitive chemoradiation or perioperative chemotherapy.
Locoregional (cT2 or node-positive to cT4a)	Tumor ≥3 cm, node-positive, or grown into the third layer of esophagus wall	Preoperative chemoradiation followed by esophagectomy (non-cervical esophagus), or definitive chemoradiation (cervical esophagus). After chemoradiation, further treatment based on disease status.	Definitive chemoradiation if not opting for esophagectomy, or palliative care.
Locoregional (cT4b)	Tumor grown through layers into nearby structures	Definitive chemoradiation. Chemotherapy alone might be possible in specific cases. After chemoradiation, further treatment based on disease status.	Palliative radiation therapy or best supportive care.

Recurrence in esophageal carcinoma

Recurrence in esophageal carcinoma can manifest in various ways. In squamous cell carcinoma, it may involve reappearance within the esophagus or as fresh carcinoma in another organ, termed locoregional recurrence. This recurrence often encompasses the esophagus and nearby lymph nodes. Treatment options for locoregional recurrence depend on the patient's history. If there was no prior chemoradiation but surgery was performed previously, choices include chemoradiation (preferred), surgery, chemotherapy, palliative care, and best supportive care. On the other hand, if there was no surgery but prior chemoradiation, the options vary based on tumor resectability and patient fitness. In cases of metastatic disease, the approach is personalized based on the patient's performance status (PS). For PS 0, 1, or 2, systemic therapy with palliative and best supportive care is considered, whereas for PS 3 or 4, priority is given to palliative and best supportive care. Systemic therapy for both squamous cell carcinoma and adenocarcinoma is contingent on performance status, with considerations for testing microsatellites, PD-L1, and human epidermal growth factor receptor 2 (HER2) before initiation, particularly when the respective types of carcinoma are suspected. Biosimilars, FDA-approved drug analogs used identically in dosage and administration, are also discussed. For managing unresectable, recurrent, or metastatic squamous cell carcinoma, preferred options differ based on HER2 expression, involving various combinations of fluoropyrimidines, oxaliplatin, cisplatin, and targeted therapy (trastuzumab). In the case of adenocarcinoma, the management options mirror those for squamous cell carcinoma but are tailored based on HER2 expression and type [19,23,24].

Advancements and emerging therapies

Significant strides in Esophageal Cancer Management have notably elevated patient outcomes. Notably, the five-year survival rates have doubled from 1995-1999 to 2011-2014, attributed to a combination of therapies and enhanced early detection methods. Early-stage esophageal cancer management has been transformed by endoscopic techniques guided by classifications like the Paris classification. Neoadjuvant chemoradiotherapy has emerged as a superior approach for locally advanced diseases. The FLOT (fluorouracil, leucovorin, oxaliplatin, and docetaxel) regimen challenges traditional approaches, demonstrating impressive survival outcomes and high pathological complete response rates. Ongoing Phase III RCTs are evaluating multimodal therapy versus chemotherapy and radiation to ascertain the optimal treatment approach, particularly for squamous cell carcinoma. Immunotherapies and targeted therapies, such as trastuzumab, are revolutionizing treatment paradigms, displaying promise in both neoadjuvant and adjuvant settings. The 'watch and wait' approach post neoadjuvant therapy is emerging, potentially avoiding immediate surgery and allowing a more tailored approach [25-28].

Significant progress has been achieved in the field of targeted therapies for esophageal cancer. The epidermal growth factor receptor (EGFR) pathway stands as a major target, with drugs like cetuximab, nimotuzumab, erlotinib, and gefitinib effectively inhibiting EGFR signaling. Strategies involving drug combinations and targeted drug delivery are being explored to enhance efficacy and mitigate toxicity. The HER2 pathway represents another pivotal target, with drugs like trastuzumab and lapatinib exhibiting efficacy in treating esophageal cancer. The vascular endothelial growth factor (VEGF) pathway, central to angiogenesis, is also a prime target, with drugs like bevacizumab and ramucirumab demonstrating potential [25-29].

Furthermore, various advances in esophageal cancer treatment have emerged. Monoclonal antibodies such as cetuximab, nimotuzumab, Sym004, PAN immunotoxin, and antibody-drug conjugates exhibit promise in inhibiting EGFR signaling. Small molecule tyrosine kinase inhibitors (TKIs) like gefitinib and afatinib effectively inhibit cell proliferation and overcome resistance. Drugs targeting HER2 and VEGF, such as trastuzumab and bevacizumab, respectively, are standard treatments for advanced esophageal cancer. Immunotherapies, particularly immune checkpoint inhibitors like PD-1/PD-L1 inhibitors and CTLA-4 inhibitors, show significant potential in treating esophageal cancer by enhancing immune responses [25-29].

Tumor vaccines and immunotherapeutic approaches hold promise in augmenting anticancer responses and inhibiting tumor progression. Various treatment paradigms, including cell-based adoptive immunotherapy and microbial ecosystem-targeted therapy, are being explored, offering the potential for revolutionizing esophageal cancer treatment [25-27,29].

Prevention and early detection

Prevention and early detection of esophageal cancer require a comprehensive approach. In primary prevention, ceasing tobacco smoking is crucial due to its carcinogenic compounds, while dietary adjustments like reducing hot beverages and processed meats, and increasing intake of fruits, vegetables, and curcumin with their micronutrients and antioxidants, can deter carcinogenesis. Abstaining from alcohol is recommended due to acetaldehyde content, which synergizes with tobacco smoke to enhance carcinogenicity. In secondary prevention, high-risk groups should undergo screenings for ESCC through methods like narrow-band imaging endoscopy, chromoendoscopy, and autofluorescence imaging. Similarly, individuals with chronic gastroesophageal reflux disease (GERD) symptoms and multiple risk factors should consider endoscopic screening for BE as part of screening for EAC, with subsequent surveillance and eradication therapy if necessary. Tertiary prevention involves post-treatment surveillance for both ESCC and EAC, aiming to detect local recurrence, metachronous ESCC, second primary cancers, and residual BE through regular examinations and interventions like anti-reflux surgery for prevention [5,30-32].

In summary, primary prevention emphasizes lifestyle adjustments like smoking cessation, a healthy diet, and limiting alcohol consumption. Secondary prevention involves targeted screening for high-risk individuals to detect early signs of esophageal cancer. Tertiary prevention focuses on post-treatment surveillance to monitor for recurrence and prevent further progression or development of secondary cancers. Additionally, intervention studies highlight the benefits of micronutrient supplementation and physical activity, while endoscopic screening and early intervention prove effective in reducing esophageal cancer mortality and incidence rates [5,30-32].

## Conclusions

Esophageal cancer presents a significant global health threat, particularly affecting males with its aggressive nature and high mortality rates. While strides have been made in the management of this disease, it's crucial to acknowledge the documented limitations in treatment modalities and palliative methods, such as postoperative complications in surgery, chemotherapy's toxic effects, and the potential for recurrence in radiation therapy. Palliative approaches, while offering symptom relief, do not provide a cure and can be challenged by the disease's progressive nature. Recognizing these constraints emphasizes the need for ongoing research and innovation to enhance patient outcomes. Promising developments in targeted therapies and immunotherapies underscore the value of personalized strategies based on the cancer's stage and location. These innovations provide hope for improved outcomes and underscore the importance of continued research and the exploration of novel treatments in addressing the rising prevalence of esophageal cancer.
